# El paciente con estenosis aórtica severa hospitalizado

**DOI:** 10.47487/apcyccv.v6i3.511

**Published:** 2025-09-24

**Authors:** Frank W. Britto

**Affiliations:** 1 Servicio de cardiología clínica, Instituto Nacional Cardiovascular - INCOR - EsSalud, Lima, Perú. Servicio de cardiología clínica Instituto Nacional Cardiovascular - INCOR - EsSalud Lima Perú

*Sr. Editor:*

Al trabajar en un centro de referencia nacional, en especial para cirugía cardiaca, lo que parece poco prevalente, en comparación con la cardiopatía isquémica, se hace prevalente y, más aun, cuando en el mundo la epidemiología cardiovascular empieza a cambiar. Es así que las enfermedades no ateroescleróticas, como las arritmias cardiacas, el tromboembolismo venoso y las valvulopatías, en especial la estenosis aórtica (EAo), se vuelven más prevalentes ^1^^,^^2^. Ante este cambio epidemiológico progresivo, se vuelve necesario contar con conductas terapéuticas definidas para enfrentar la nueva demanda.

Las Guías del American Collegue of Cardiology/American Heart Association (ACC/AHA) para el manejo de las enfermedades valvulares, desde 2014 hasta la actualidad, clasifican a la EAo en cuatro etapas ^3^:

Etapa A: en riesgo: pacientes con válvula aórtica bicúspide, esclerosis aórtica.

Etapa B: progresiva; EAo leve a moderada.

Etapa C: EAo severa asintomática.

C1: con fracción de eyección del ventrículo izquierdo (FEVI) ≥ 50%.

C2: con FEVI < 50%.

Etapa D: EAo severa sintomática.

D1: pacientes en clase funcional de la New York Heart Association (NYHA) II (se refiere a la clase funcional y no solo a la disnea).

D2: pacientes con bajo flujo, baja gradiente media (GM) y FEVI reducida (velocidad máxima [Vmax] > 4 m/s, GM < 40 mmHg, FEVI < 50%).

D3: paciente con baja GM, con FEVI normal o bajo flujo paradójico (FEVI > 50%, GM < 40 y área valvular aórtica [AVA] > 1 cm^2^).

En el año 2017, P. Généraux *et al*. ^4^ presentaron una nueva clasificación, basada en el daño miocárdico generado por la EAo, dividida también en etapas y que sirvió para predecir el pronóstico al año del reemplazo de la válvula aórtica (RVAo).

Etapa 0: sin daño cardiaco.

Etapa 1: daño en ventrículo izquierdo (hipertrofia ventricular izquierda [HVI], relación E/e’ > 14 o FEVI < 50%).

Etapa 2: daño en la aurícula izquierda (AI) y/o válvula mitral (volumen de AI > 35 mL/m^2^, insuficiencia mitral o fibrilación auricular).

Etapa 3: daño a la vasculatura pulmonar y/o válvula tricúspide (presión sistólica de arteria pulmonar >/= 60 mmHg o insuficiencia tricúspidea).

Etapa 4: daño al ventrículo derecho (disfunción de ventrículo derecho).

**Figura 1:**
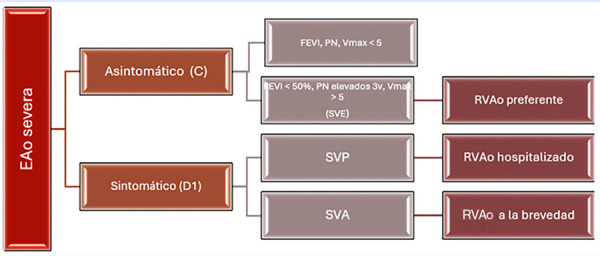
Clasificacion completa de la estenosis aortica severa.

Recientemente, el propio Généraux ^5^^,^^6^ propuso una nueva categorización clínica para personas que padecen EAo severa, la cual facilita una clasificación detallada (Figura 1).

FEVI: fraccion de eyeccion del ventriculo izquierdo. PN: peptidos natriureticos (BNP, NT-proBNP). Vmax: velocidad maxima en m/s. SVE: síndrome valvular estable. SVP: sindrome valvular progresivo. SVA: sindrome valvular agudo. RVAo: reemplazo valvular aortico (TAVI o SAVI).

1. Síndrome valvular estable (etapa C [ACC/AHA] o etapa 0,1 de la clasificación del 2017). Se caracteriza porque el paciente tiene EAo severa pero está asintomático. A su vez, tiene dos grupos:

• El que no tiene algún marcador de riesgo (FEVI normal, Vmax, proBNP). Requiere seguimiento cercano, antes de los 6 meses. En el grupo conservador o de vigilancia del estudio EARLY TAVR ^7^, 25% de estos sujetos presentaron síntomas: 60% progresivos y 38% síntomas avanzados (NYHA III/IV, edema agudo de pulmón, síncope, arritmia ventricular o fueron recuperados de muerte súbita).
• Asintomático pero con criterios de alto riesgo: Vmax > 5 m/seg ^8^ y/o FEVI < 50% y/o BNP > 100 pero < 400 o NT - proBNP > 1000 pero < 1500 ^9^. En este caso, la recomendación es el reemplazo valvular aórtico (RVAo) electivo.

2. Síndrome valvular progresivo (etapa D1 [ACC/AHA] o etapa1 de la clasificación del 2017). Son pacientes en clase funcional NYHA II, caracterizados por disnea, mareos, angina y fatiga (la disfunción diastólica produce congestión pulmonar) y edema de miembros inferiores. El tratamiento es con diuréticos y RVAo hospitalizado.

3. Síndrome valvular agudo (etapa D1 [ACC/AHA], etapas 2-4 de la clasificación del 2017). Son pacientes en NYHA III/IV, que se presentan con síncope o falla cardiaca descompensada. Además, se caracterizan por reducción de la FEVI > 10%, BNP o NT-proBNP > 3 veces el límite superior para la edad, fibrilación auricular, angina clase funcional canadiense 3-4, hipotensión arterial, *shock*, arresto cardiaco, endocarditis o que ha sido recuperado de muerte súbita.

El síncope en el paciente con EAo es causado por bloqueo AV, bajo gasto al esfuerzo, pero principalmente vasovagal, explicado porque existen mecanorreceptores localizados en la pared ínfero-lateral endocárdica del VI, cuyos impulsos viajan por vías aferentes a la médula, resultando en la inhibición del tono simpático y la estimulación parasimpática (bradicardia e hipotensión). Estos mecanorreceptores responden al incremento de presión sistólica. La isquemia inferolateral subendocárdica también produce el mismo resultado ^10^.

En el tratamiento del paciente con EAo y falla cardiaca descompensada se recomienda: ^(^^11^^-^^15^

• Ante normotensión arterial, usar nitroprusiato con diuréticos ^15^.
• En hipotensión arterial o shock cardiogénico.

1. Vasopresores/inotrópicos (evitando la frecuencia cardiaca > 100/min).

2. Manejar factores precipitantes:

a. Fibrilación atrial (controle ritmo y/o frecuencia).

b. Anemia.

c. Sepsis (la ausencia de bacteriemia permite el RVAo).

3. Diferir en lo posible la intubación orotraqueal por el alto riesgo de empeoramiento periintubación (caída de la presión de perfusión coronaria, empeoramiento de la isquemia o empeoramiento del *shock*).

4. Evaluar futilidad versus soporte cardiaco mecánico temporal.

5. Evaluación completa con imágenes: ecocardiografía transtorácica y transesofágica, cateterismo izquierdo y derecho (mantenga CAP) y tomografía cardiaca con protocolo TAVI.

6. RVAo a la brevedad.
